# Supplementary material for: The interactome of intact mitochondria by cross-linking mass spectrometry provides evidence for coexisting respiratory supercomplexes
Source: Mol Cell Proteomics. 2017 Dec 8;17(2):216–32. doi: 10.1074/mcp.RA117.000470 (PMC5795388; doi:10.1074/mcp.RA117.000470)
Supplement: Supplemental Data [file supp_RA117.000470_133922_0_supp_23978_fzffwf.zip › spectra_annotation/mito_spectra_annotation/497-1-10-1-12-1.pdf]

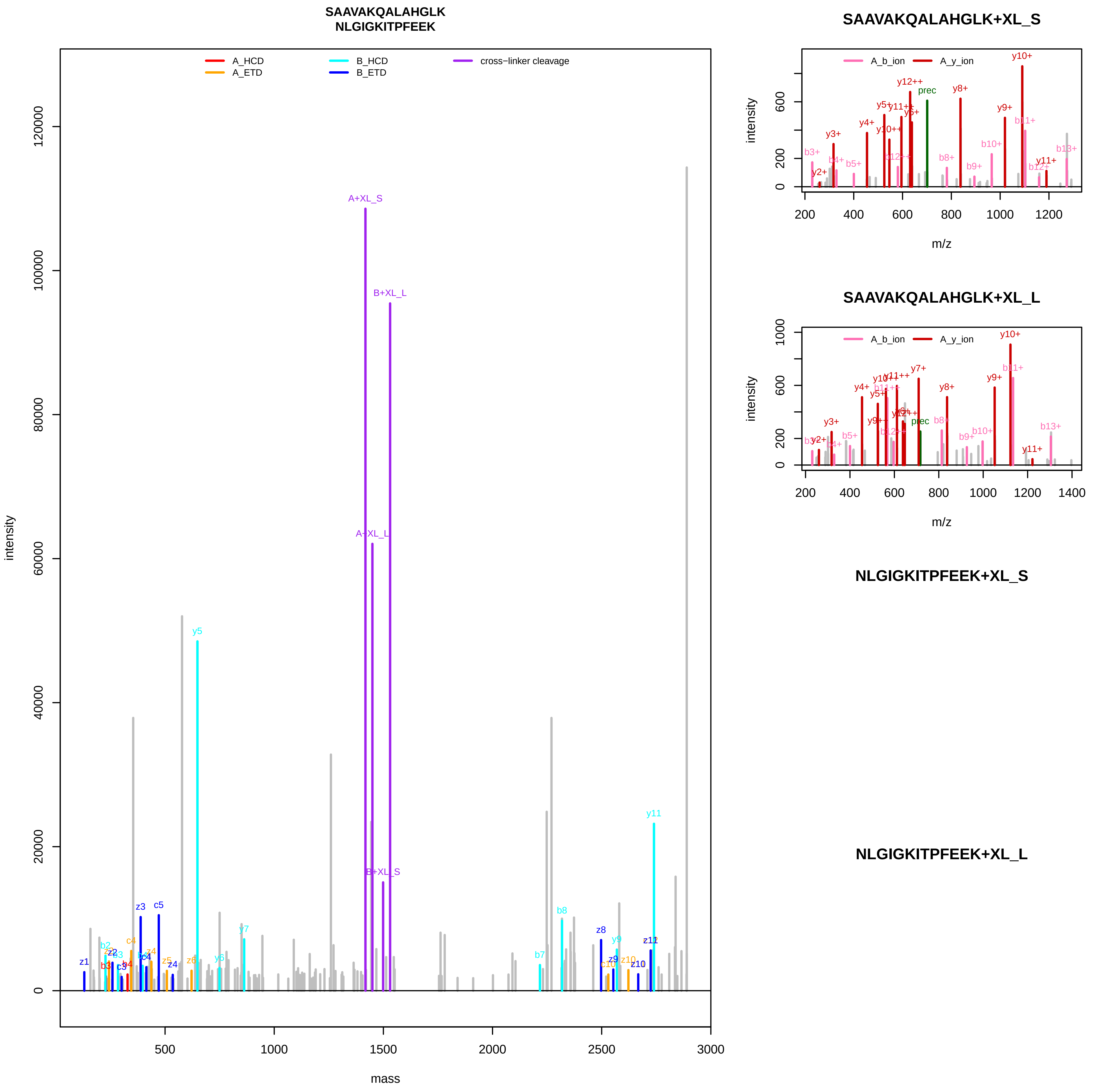

SAAVAKQALAHGLK
NLGIGKITPFEEK
A_HCD
A_ETD
B_HCD
B_ETD
cross−linker cleavage
A+XL_S
B+XL_L
A+XL_L
B+XL_S
y5
y6
y7
y11
b7
b8
y9
z1
z2
z3
c3
c4
c5
z4
z8
z9
z10
z11
c10
z10
intensity
mass
SAAVAKQALAHGLK+XL_S
A_b_ion
A_y_ion
prec
m/z
SAAVAKQALAHGLK+XL_L
NLGIGKITPFEEK+XL_S
NLGIGKITPFEEK+XL_L
